# Fluorescence colocalization microscopy analysis can be improved by combining object‐recognition with pixel‐intensity‐correlation

**DOI:** 10.1002/biot.201600332

**Published:** 2016-07-26

**Authors:** Bernhard Moser, Bernhard Hochreiter, Ruth Herbst, Johannes A. Schmid

**Affiliations:** ^1^ Dept. of Vascular Biology and Thrombosis Research, Center for Physiology and PharmacologyMedical University of ViennaViennaAustria; ^2^ Inst. of Immunology, Center for Pathophysiology, Infectiology and ImmunologyMedical University of ViennaViennaAustria

**Keywords:** Colocalization, Fluorescence, ImageJ, Image processing, Microscopy

## Abstract

The question whether two proteins interact with each other or whether a protein localizes to a certain region of the cell is often addressed with fluorescence microscopy and analysis of a potential colocalization of fluorescence markers. Since a mere visual estimation does not allow quantification of the degree of colocalization, different statistical methods of pixel‐intensity correlation are commonly used to score it. We observed that these correlation coefficients are prone to false positive results and tend to show high values even for molecules that reside in different organelles. Our aim was to improve this type of analysis and we developed a novel method combining object‐recognition based colocalization analysis with pixel‐intensity correlation to calculate an object‐corrected Pearson coefficient. We designed a macro for the *Fiji*‐version of the software *ImageJ* and tested the performance systematically with various organelle markers revealing an improved robustness of our approach over classical methods. In order to prove that colocalization does not necessarily mean a physical interaction, we performed FRET (fluorescence resonance energy transfer) microscopy. This confirmed that non‐interacting molecules can exhibit a nearly complete colocalization, but that they do not show any significant FRET signal in contrast to proteins that are bound to each other.

**Abbreviations**
ANOVA: analysis of variances
DMEM: Dulbecco's modified essential medium
ECFP: enhanced cyan fluorescent protein
ER: endoplasmic reticulum
EYFP: enhanced yellow fluorescent protein
FBS: fetal bovine serum
FRET: fluorescence resonance energy transfer
HEK: human embryonic kidney cells
ICQ: intensity correlation quotient
JACOP: Just Another Colocalization Plugin (for ImageJ)
NA: numerical aperture

## 1 Introduction

In the last two decades, we have seen an incredible progress in fluorescence microscopy techniques and in parallel a significant advance in methods to label proteins genetically by fusing them to spectrally distinct fluorescent proteins. This allowed addressing central issues in cell biology and life sciences, such as the question, whether a certain protein localizes to a specific subcellular compartment or whether it shares the same localization with another molecule of interest. Answering these questions is often essential to clarify cellular processes as they are frequently regulated by macromolecular interactions or by specific localizations of molecules within the complex compartmental structure of eukaryotic cells. Since discrimination of different fluorescence colors is rather easy with standard microscopy equipment, many scientists use fluorescence colocalization as an indication that a molecule localizes to a certain compartment or that two molecules interact with each other. However, in particular the latter assumption is often an over‐interpretation, as the occurrence of two molecules in the same subcellular regions does not necessarily mean that they are physically binding to each other. Sophisticated novel techniques such as superresolution microscopy address these questions with better precision 1, nevertheless they can still not discriminate unambiguously between functional interaction and incidental colocalization. Furthermore, these techniques require expensive equipment that is not easily available and rather complex to use.

In many cases, the methods to analyze and score microscopic colocalization are often simple and descriptive rather than quantitative. A method that is still used by many scientists is to assess the color overlay of two different fluorescent markers, with for instance green and red fluorescence resulting in a yellow color in case of colocalization. Nevertheless, such a visual evaluation requires comparable fluorescence intensities of the two markers and is obviously far from being quantitative. These issues had been recognized quite early in the development of fluorescence microscopy, which has led to the concept of calculating statistical parameters to evaluate the correlation of fluorescence‐intensities of two (or more) detection channels on a pixel‐by‐pixel basis. The underlying statistical background had already been developed in 1896 by Pearson 2, but only about hundred years later, Manders et al. proposed Pearson's correlation coefficient as a more quantitative measure of colocalization based on the pixel‐intensity correlation of two fluorescence channels 3. This concept has been followed up by the same group, suggesting two coefficients *M1* and *M2*, which describe the extent of the fluorescence of colocalizing objects relative to the total fluorescence in channel 1 and 2, respectively 4. Later, the intensity‐correlation based methods have been further elaborated by Li et al., who compared the pixel intensity of a detection channel with (*A*_i_ − *A*_mean_) × (*B*_i_ − *B*_mean_), where *A*_i _and *B*_i_ are individual intensities of a pixel in channels A and B, while *A*_mean_ and *B*_mean_ are the mean intensities 5. This provided improved possibilities for evaluation and visualization of colocalization. An alternative concept has been followed by Costes et al., who developed a method for automated and unbiased threshold determination 6, solving some of the issues that occur with the method of Manders et al., as the latter is quite sensitive to background, which has to be subtracted manually 4. A number of elegant review articles discuss theory and practice of colocalization analysis and explain the different parameters in more detail 7, 8, 9, 10, 11, 12, 13. Many of the different methods have been incorporated in various commercial image analysis programs, but also as plugins into powerful free software such as *ImageJ* from the National Institute of Health, USA. A comprehensive tool for quantitative colocalization analysis is an *ImageJ* plugin termed *JACoP* (for *just another colocalization plugin*; 7) and a similar sophisticated feature, designated as *coloc2* is part of the analysis options of the expanded *ImageJ* version *Fiji*. The *JACoP* plugin goes already slightly beyond pixel‐intensity based correlation by performing some object‐based colocalization analysis using the calculation of distances between centers of mass or coincidences of thresholded objects. However, it only counts apparently colocalizing objects in comparison to total objects, which can vary substantially if the number of objects is low.

Our aim was to compare different methods of colocalization analysis and to improve the reliability by combining pixel‐intensity correlation with an object‐based method that quantifies the area fraction of colocalization. Furthermore, we intended to complement colocalization analysis with FRET microscopy, which gives positive signals just in case two fluorescent molecules are closer than about 10 nm, thereby reporting only real physical interaction rather than random colocalization. This method relies on fluorescence resonance energy transfer from a donor fluorophore to an acceptor fluorophore (with a longer excitation and emission wavelength) via a dipole interaction leading commonly to a decrease in donor emission and an increase in acceptor fluorescence 14, 15, 16, 17, 18, 19, 20. While the physical background of this phenomenon is quite complex, the technical realization is rather simple and can be performed on standard fluorescence microscopes.

## 2 Materials and methods

### 2.1 Transfection of cells with markers of subcellular compartments

HEK293T cells were cultivated in DMEM medium with 10% FBS. For microscopy, cells were transferred onto Ibidi ibiTreat eight‐well slides (ibidi GmbH, Am Klopferspitz 19, 82152 Planegg/Martinsried; cat# 80826) two days before measurement. One day after, cells were transfected at ~70% confluency with organelle markers, using ThermoFisher Scientific Turbofect transfection reagent (Cat# R0531) according to product information. Transfected cells were incubated overnight, and medium was exchanged at least 1 h prior to microscopic measurement. Organelle markers were from Clontech Laboratories, Inc. (Mountain View, CA, USA) and comprised the following vectors:

- pEYFP‐Mito and pECFP‐Mito (mitochondria); containing a mitochondrial targeting sequence derived from the precursor of subunit VIII of human cytochrome c oxidase
- pEYFP‐Mem and pECFP‐Mem (membranes); containing the Neuromodulin N‐terminal 20 amino acid sequence for cytoplasmic membrane targeting.
- pEYFP‐ER and pECFP‐ER (endoplasmic reticulum); containing the ER targeting sequence of calreticulin.
- pECFP and pEYFP: localizing to cytosol and nucleus (diffusing through the nuclear pore).

### 2.2 Confocal laser scanning microscopy

Confocal laser scanning microscopy was performed with an A1 R+ system from Nikon with a 12‐bit intensity range one or two days after cell transfection.

The Nikon system employed a Ti microscope with a 60× plan apochromatic oil immersion objective (NA1.4). Excitation was done with an Ar‐laser (457 nm for ECFP and 514 nm for EYFP in sequential mode) and detection with a pinhole value of 35.8. A 400–457/514 nm dichroic mirror was used; ECFP‐emission was recorded with a 482/33 nm filter and EYFP‐emission with a 540/30 nm filter with a line averaging of 4 or 8.

These imaging settings have been verified to discriminate clearly between ECFP and EYFP.

### 2.3 FRET‐microscopy

FRET images were taken on the Nikon A1 confocal laser‐scanning microscope as described above, using a three‐filter‐configuration based approach 18, 20. DONOR‐fluorescence (ECFP emission at ECFP excitation; ECFP‐channel) was obtained with a 403 nm laser and a 482/35 (465–500 nm) filter cube. Raw‐FRET fluorescence (acceptor = EYFP emission at donor excitation) was acquired with a 403 nm laser and a 540/30 filter cube (525–555 nm); ACCEPTOR‐fluorescence (EYFP emission at EYFP excitation; EYFP‐channel) was captured with a 514 nm laser and a 540/30 filter cube (525–555 nm). Pure ECFP and EYFP were used for determination of bleed‐through factors *df* and *af*.

df=rawFRET signal of donor alone/DONOR(ECFP)

af=rawFRET signal of acceptor alone/ACCEPTOR(EYFP)

A corrected FRET‐image (*cFRET*) according to Youvan et al. 21 was determined by subtracting the product of the bleed‐through factors and the corresponding donor and acceptor channel from the raw FRET channel of the sample as follows:

cFRET=rawFRET−(DONOR×df)−(ACCEPTOR×af)

FRET efficiency was calculated as suggested by Feige et al. 22 as follows:

$\mathrm{FRET}-\mathrm{Eff}.\;=\text{1}-\;\frac{\mathrm{DONOR}}{\mathrm{DONOR}+\mathrm{cFRET}}$

Based on the assumption that the *cFRET* signals equals the fluorescence increase of the donor after complete bleaching of the acceptor and the known correlation 20:

$\mathrm{FRET}-\mathrm{Eff}.\;=\text{1}-\;\frac{\mathrm{DONOR}\;\mathrm{in}\;\mathrm{presence}\;\mathrm{of}\;\mathrm{ACCEPTOR}}{\mathrm{DONOR}\;\mathrm{in}\;\mathrm{absence}\;\mathrm{of}\;\mathrm{ACCEPTOR}}$

Analysis was done in *ImageJ*, using a self‐developed macro for pixel‐per‐pixel analysis of acquired images.

### 2.4 Image processing and calculation of a combined colocalization coefficient

Images were imported into the *Fiji* version (http://fiji.sc) of the free image processing software *ImageJ*. *Fiji* contains a number of pre‐installed plugins including a procedure for colocalization analysis, designated as *coloc2*, which calculates a variety of colocalization parameters such as the Pearson coefficient, Spearman's rank correlation, Manders correlation and the ICQ value as suggested by Li 5, all of which are based on pixel‐intensity‐correlation measurements but do not include object‐recognition approaches. We designed a macro, which combines the *coloc2* plugin with an object‐based colocalization analysis (as specified in the Supporting information). This macro subtracts the background of both channels after thresholding of signal‐containing areas with the ”triangle“ algorithm of *Fiji*. The channels are equalized to the intensity range to compensate for potential intensity differences between the channels. Thereafter, the *coloc2* plugin is called from within the macro, employing a bisection threshold regression without a region of interest or mask. Numerical correlation parameters are recorded, as well as the 2D intensity histogram for visualization of the correlation between the two channels. Subsequently, the macro performs an object identification, applying a ”MaxEntropy“ threshold algorithm of *Fiji*. Since a proper thresholding between objects and background depends very much on the nature of the stained objects and the conditions of image acquisition, we also created a macro allowing *Default* thresholding and a macro with manual thresholding for both channels (see Supporting information online). In all macro versions, thresholded objects are binarized followed by a watershed segmentation to separate adjacent entities. Using the image calculator and the ”Max“ algorithm, a combination of the objects of the two channels is calculated; by employing an ”AND“ operand, colocalizing regions are determined. The ”Analyze Particles“ feature of *Fiji* is applied with a minimum area of 25 pixels to calculate numbers and total areas of identified objects; their combination and the colocalizing regions. The result values recorded by the macro were copied to MS‐Excel 2013 to calculate the fraction of colocalization as compared to the combination of thresholded objects in the two channels. The results of *coloc2* derived intensity‐based correlation analysis were copied into the same MS‐Excel sheet and the different parameters, such as the Pearson coefficient, Li's ICQ value or the Manders coefficients were multiplied with the colocalization fraction value. This results in a reduction of the pixel intensity‐based correlation according to the percentage of object‐based colocalization.

## 3 Results and discussion

### 3.1 Intensity based colocalization analyses result in high correlation values for non‐colocalizing compartments

**Figure 1:**
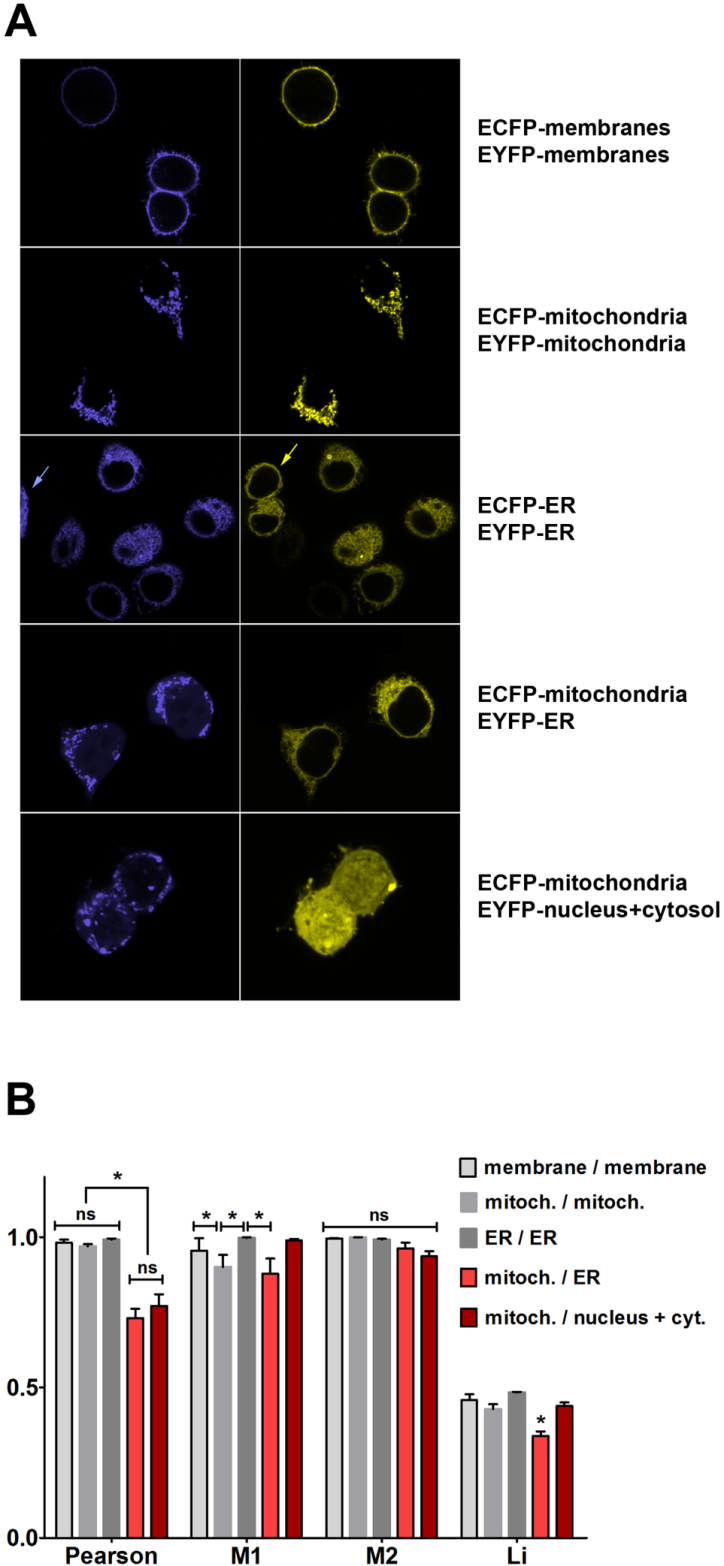
Confocal laser scanning microscopy of various subcellular compartments and classical colocalization analysis. (**A**) Representative confocal microscopy images of HEK293T cells transfected with expression plasmids coding for ECFP‐ and EYFP‐tagged cellular compartment markers localizing to the cytoplasmic membrane, mitochondria, ER or nucleus plus cytosol, as indicated next to the images. Microscopy was done with a Nikon A1 laser scanning microscope using a 60× oil immersion objective (NA 1.4). Complete colocalization is observed in the samples expressing ECFP‐ and EYFP‐tagged markers for the same compartment (upper three rows), whereas distinct localization is observed for the combination of ECFP‐mitochondrial marker with either an EYFP‐tagged ER marker or EYFP alone, which localizes to nucleus and cytosol (lower two rows). The yellow and the blue arrow indicate cells that are only labeled with one marker thereby verifying the selectivity of the detection. (**B**) Classical colocalization analysis of images transfected with compartment markers as in (**A**) using the *coloc2*‐plugin of the extended *ImageJ* version *Fiji*. The Pearson's R value was computed, as well as Manders' coefficients (*M1* and *M2*) and the ICQ value according to Li as indicated (data represent mean +/– standard error of mean, *n* = 6, asterisks indicate a significant difference with *p* < 0.05; ”ns“ stands for ”not significant“; not specifically labeled columns do not exhibit significant differences from the other unlabeled columns). Analysis of variances (ANOVA) was performed followed by a Tukey's multiple comparisons test with GraphPad Prism 6.0.

In a previous project 23, we had noticed that molecules, which exhibited clearly different localizations in the cell as assessed by fluorescence microscopy, still gave high numerical colocalization values with intensity‐based coefficients such as Pearson's or Manders' correlation parameters. Evaluating all the relevant pixel‐intensity based parameters with the *ImageJ* plugin *JACoP* revealed that they were prone to false positive values or rather high standard deviations, which prompted us to develop an object‐based colocalization analysis technique that scores the area fraction of colocalization. Here, we extended this study by using organelle markers with known subcellular localization and a systematic evaluation of classical colocalization coefficients. Human cells transfected with ECFP‐ and EYFP‐tagged markers for cytoplasmic membranes, mitochondria, ER or transfected with EYFP alone, which spreads out through cytosol and nucleus, revealed the expected localization pattern in high‐resolution confocal laser‐scanning microscopy (Fig. 1A) with a clear separation of the two spectrally different markers. Next, we used these images for colocalization analyses applying the *coloc2* measurement option of the image analysis software *Fiji*, which delivers all the important intensity correlation parameters. As expected, we found very high correlation values, when the same compartments were labeled with ECFP and EYFP (Fig. 1B). However, even non‐colocalizing markers that stain different subcellular compartments showed high numerical values of intensity correlation. Manders' coefficient *M2* did not show any statistically significant difference between the distinctly stained cells and also *M1* as well as the intensity correlation quotient according to Li et al. failed to distinguish unambiguously between colocalizing and non‐colocalizing markers 5. ANOVA and Tukey's multiple comparisons test revealed only partial significance for the distinction of samples. The Pearson's correlation coefficient turned out to be the best discriminator between known colocalizing and non‐colocalizing compartments in this systemic comparison, although it also showed a quite high numerical value of intensity correlation. From these data, it can be concluded that classical intensity‐based methods fail to provide a definite answer, whether different fluorescent markers colocalize in the cell.

### 3.2 Normalizing intensity correlation coefficients with the percentage of colocalizing objects provides a more robust assessment of colocalization

**Figure 2:**
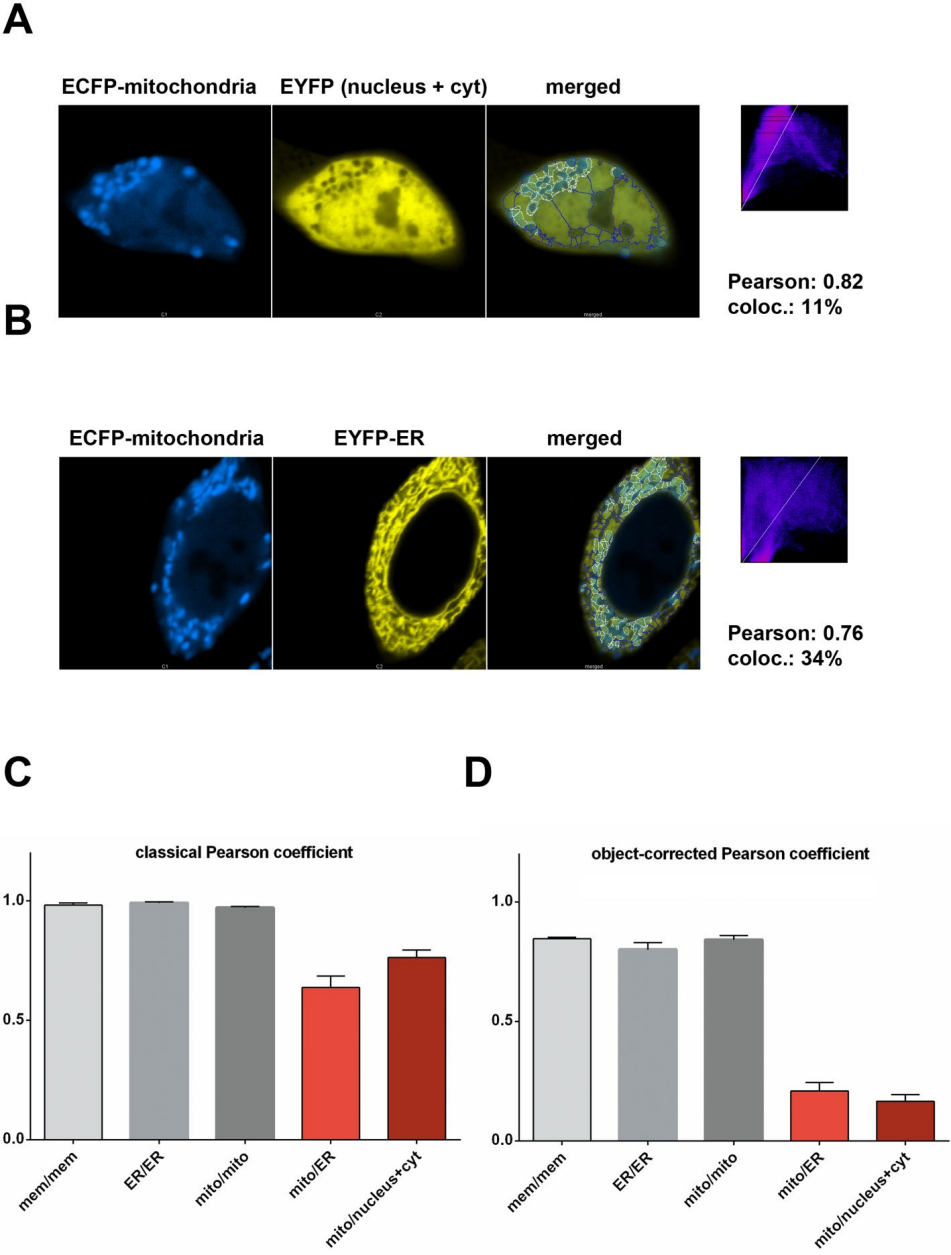
Fluorescence colocalization analysis combining pixel‐intensity correlation with object‐recognition. (**A**) Laser scanning microscopy of HEK293T cells transfected with an ECFP‐tagged marker for mitochondria and EYFP localizing to cytosol and nucleus as indicated. A *Fiji/ImageJ* macro (as specified in the Methods section) was used for object recognition, segmentation and calculation of a combination, as well as a colocalization mask. Blue lines in the merged image indicate the combination of objects above a MaxEntropy threshold, white lines indicate colocalizing thresholded objects. Right panel: 2D intensity histogram output of *coloc2* analysis performed with Fiji/ImageJ. The text indicates the classical Pearson coefficient of the pixel‐intensity correlation, as well as the percentage of colocalization compared to the combination area. (**B**) Laser scanning microscopy of HEK293T cells transfected with an ECFP‐tagged marker for mitochondria and an EYFP‐tagged marker for the ER and analysis as described in (**A**). (**C**) Calculation of the classical Pearson coefficient of colocalization for different organelle markers (as shown in Fig. 1 and Fig. 2A and 2B). Grey columns indicate cells transfected with equal compartment markers tagged with ECFP and EYFP (abbreviations: mem, membranes; mito, mitochondria; cyt, cytosol). Red columns indicate cells transfected with marker localizing to different organelles (mean +/– standard error of mean, *n* = 6). (**D**) Calculation of an object‐corrected Pearson coefficient by multiplying the classical Pearson coefficient with the fraction of colocalizing objects above the threshold (mean +/– standard error of mean, *n* = 6).

Since we found that statistical methods of pixel‐intensity correlation do not provide a clear evaluation of colocalization and based on our previous observations that object‐recognition based strategies seem to be superior 23, we thought of combining both approaches. To that end, we designed a macro for the *Fiji* software package, which automates a series of image processing steps that can be executed in batch mode for a whole series of images in a folder. In brief, this macro performs unbiased background subtraction and normalization of intensity ranges followed by classical colocalization analysis with the embedded *coloc2* routine. Afterwards, objects are identified with a threshold algorithm, and adjacent objects are separated with the ”watershed“ function of the program. Objects of the two channels are then superimposed to generate a combination mask. Furthermore, the overlap is determined using a Boolean ”AND“ operand to quantify number and area of colocalizing objects. A summary of thresholded objects, the combination and the colocalization area is calculated and recorded in the results window of the software. In parallel, the *coloc2* routine provides an output in the Log‐window, as well as in a specific output box showing a 2D histogram of intensity correlations and the numerical correlation coefficients. We copied all these parameters into a spreadsheet program (MS‐Excel™), where we calculated the percentage of colocalizing objects and weighted the intensity correlation parameters with the fraction of colocalization. Since we found that the classical Pearson's coefficient provided a better discrimination than the other parameters, we calculated an ”object‐corrected Pearson coefficient“ as primary read‐out of our combined colocalization analysis method. Testing this approach with cells transfected with markers localizing to different compartments revealed a clear improvement of the analysis over classical intensity‐correlation methods (Fig. 2). While the classical Pearson's coefficient was quite high for the comparison of mitochondria with either an ER‐marker or a marker localizing to nucleus and cytosol, the object‐corrected Pearson coefficient provided a robust discrimination from truly colocalizing samples (Fig. 2B). To verify the robustness of our analysis algorithm, we analyzed a second, completely independent dataset and obtained similar results (Supporting information, Fig. S1). In the course of our study, we noticed that different cellular structures and stainings require distinct thresholding algorithms for a reliable automated object recognition. *Fiji* and *ImageJ* provide a panel of pre‐installed algorithms using diverse histogram analysis routines to separate into background and objects. The default threshold option seemed appropriate for us in cases, where a spatially homogenous staining over a dark background is observed, while a ”MaxEntropy“ threshold seemed better suited for fine structures such as ER or when the specific staining was observed on top of a rather unspecific cellular background above the image background. Since the thresholding requirements depend very much on the specific nature of the images, we decided to design in addition a macro allowing a manual thresholding of the two channels. However, we think that the advantage of an unbiased, automated thresholding routine is lost in this case requiring a fair and objective threshold adjustment by the user.

### 3.3 Molecular interactions cannot be claimed from complete colocalization and need to be verified by alternative methods like FRET microscopy

**Figure 3:**
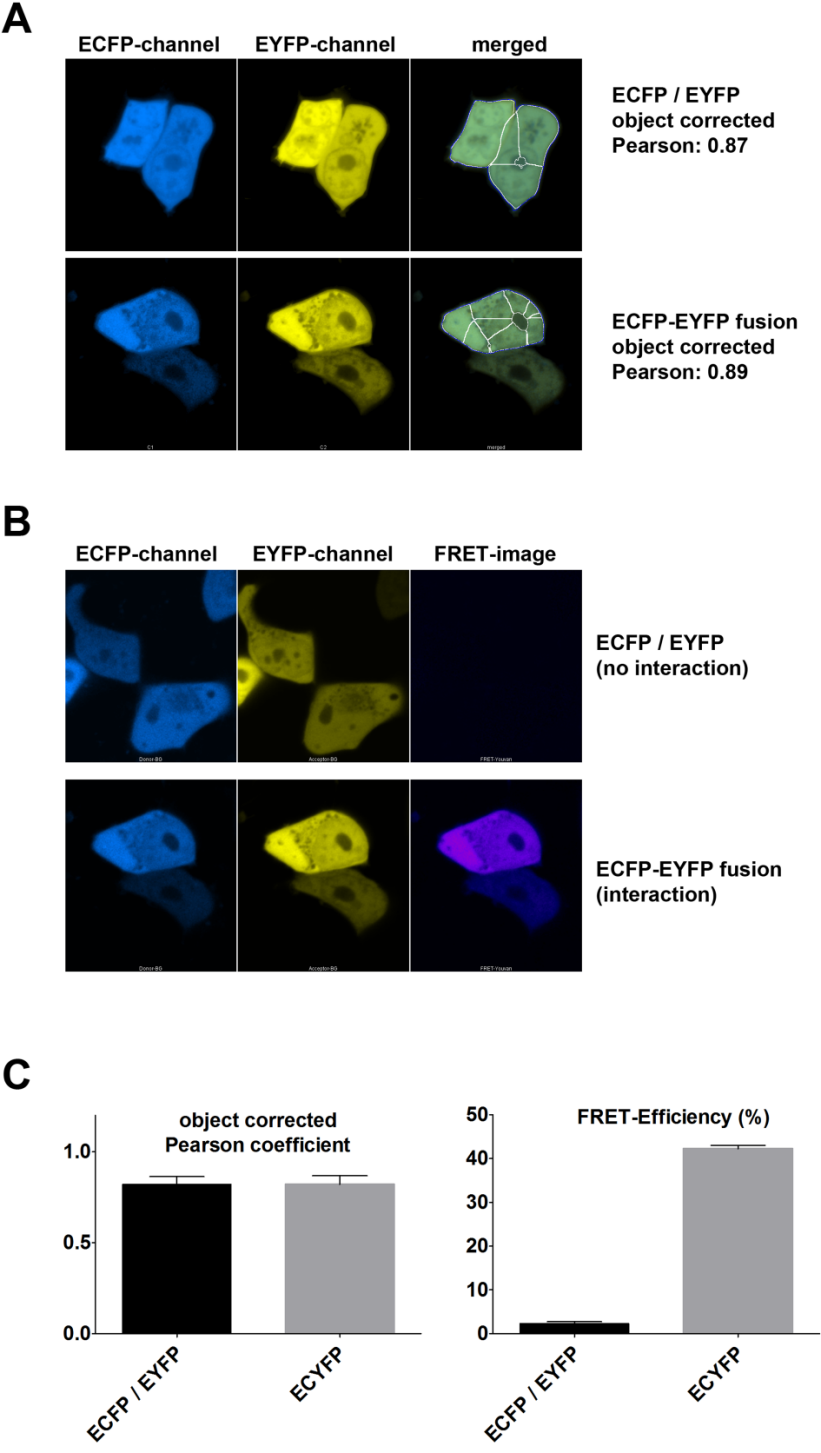
FRET microscopy as final proof of proximity of molecules. (**A**) Laser scanning microscopy of HEK293T cells transfected with ECFP and EYFP that do not interact with each other, but which localize to the same compartments (upper panel) or transfected with an ECFP‐EYFP fusion protein, where the two fluorophores are in close proximity (about 5.3 nm, lower panel). An object‐corrected Pearson correlation coefficient was calculated as described in Fig. 2 and in the text. Blue lines in the merged image indicate the combination of objects above a MaxEntropy threshold, white lines indicate colocalizing thresholded objects. Due to the threshold algorithm the lower expressing cell is not considered in the lower panel. (**B**) Laser scanning FRET microscopy of HEK293T cells transfected as in (**A**). A corrected FRET image has been calculated as described in the Methods section. (**C**) Left panel: Quantification of the object‐corrected Pearson coefficient for cells as transfected in (**A and B**) (mean +/– standard error of mean, *n* = 6). Right panel: Quantification of the apparent FRET‐efficiency of these cells as described in the Methods section (mean +/– standard error of mean, *n* = 6).

In many research articles, fluorescence colocalization is used to state that two molecules of interest interact with each other. While a functional connection might be plausible in case of a clear colocalization, a real physical interaction can certainly not be claimed based on a mere colocalization. This is evident for larger cellular structures such as the nucleus, which can be populated by many different types of molecules without any interaction between them. However, even for fine structures such as endosomes, lysosomes or the tubule‐reticular network of the ER, any colocalization of molecules cannot be seen as a proof of interaction, given that the limit of optical resolution is just in the range of 300 nm in standard laser scanning microscopy. While superresolution microscopy can go down to approximately 30 nm resolution, this distance is still beyond the spatial range of most protein complexes. Nevertheless, the physical phenomenon of fluorescence energy transfer (FRET) is ideally suited to monitor macromolecular binding, as it gives a signal only at a proximity that is closer than about 10 nm. Therefore, we set out to test FRET microscopy as a complementary technique to discriminate between random colocalization and real interaction of molecules. We transfected HEK293T cells with expression constructs coding for ECFP and EYFP separate from each, which show perfect colocalization in the cells and with a plasmid encoding a fusion protein of ECFP and EYFP, mimicking a molecular interaction. Analyses of microscopy images from these cells using our improved, object‐corrected colocalization macro revealed as expected a high degree of colocalization in both cases (Fig. 3A and 3C, left panel). However, visualizing the FRET effect with the three‐filter cube method as described in the Methods section did not show any significant positive signal in case of the non‐interacting pair of ECFP and EYFP, while a strongly positive signal could be observed for the ECFP‐EYFP chimera (Fig. 3B). Using a normalization algorithm, which compensates for differences in expression levels, the FRET value can be quantified for different cells allowing a statistical evaluation. This analysis revealed a clearly positive FRET efficiency value for the fusion protein in contrast to the control expressing ECFP and EYFP separate from each other. Thus, FRET microscopy and FRET efficiency calculations can answer the question, whether two proteins are in close proximity to each other, while the quantification of an object‐corrected Pearson's coefficient fails to discriminate between incidental colocalization and real association (Fig. 3C). However, it has to be stated that FRET microscopy can lead to false negative results for truly interacting molecules in case the molecular distance of the fluorophores is beyond 10 nm or the orientation of the fluorophores doesn't allow the dipole‐dipole interaction necessary for FRET.

## 4 Concluding remarks

The frequent use of colocalization analyses in microscopy asks for a robust and reliable method of quantification, which is often not achieved with the current methods of pixel‐intensity correlation. By combining the classical statistical methods with object‐recognition and calculation of a percentage of colocalizing object‐areas, we can calculate an object‐corrected Pearson coefficient representing a more robust measure of colocalization. Finally, we demonstrate that colocalization is not sufficient to claim a physical association of molecules and that the latter requires alternative methods such as FRET micros copy.

## Supporting information

As a service to our authors and readers, this journal provides supporting information supplied by the authors. Such materials are peer reviewed and may be re‐organized for online delivery, but are not copy‐edited or typeset. Technical support issues arising from supporting information (other than missing files) should be addressed to the authors.

Supporting Information
