# Supplementary material for: Fluorescence colocalization microscopy analysis can be improved by combining object‐recognition with pixel‐intensity‐correlation
Source: Biotechnol J. 2016 Jul 26;12(1):1600332. doi: 10.1002/biot.201600332 (PMC5244660; doi:10.1002/biot.201600332)
Supplement: Supplementary file 1 — Supporting Information [file BIOT-12-0-s001.pdf]

Biotechnology
Journal

Supporting Information for DOI 10.1002/biot.201600332

# Fluorescence colocalization microscopy analysis can be improved by combining object-recognition with pixel-intensity-correlation

*Bernhard Moser, Bernhard Hochreiter, Ruth Herbst, Johannes A. Schmid*

© 2016 Wiley-VCH Verlag GmbH & Co. KGaA, Weinheim

## Supplementary data

# Content

Supplementary information 1: *Fiji/ImageJ* Macro with automated *MaxEntropy* threshold ............. 1

Supplementary information 2: Fiji/ImageJ Macro with automated Default threshold ...................... 5

Supplementary information 3 *Fiji/ImageJ* Macro with manual user-defined threshold ................. 10

Suppl. Figure 1: Test of the analysis procedure with an independent data set.............................. 13

## Supplementary information 1: *Fiji/ImageJ* Macro with automated *MaxEntropy* threshold

### *Fiji – ImageJ* Macro for objected corrected colocalization analyses

### with automated *MaxEntropy* threshold

```
// The macro uses two channels (termed C1, C2) to determine colocalization
// From each channel a background is subtracted after thresholding
// with a triangel algorithm; outside the thresholded objects the image is cleared
// Then the channels are equalized to the intensity range so that the following
thresholding
// finds equal conditions for both channels (in case of different intensities)
// A MaxEntropy threshold is then used for object identification for each channel.
// Objects are binarized followed by a watershed segmentation
// overlapping objects are determined (colocalization)
// A combination of objects is generated (combination)
// area measurements of colocalization and combination serve to determine
// the fraction of colocalization

run("Set Measurements...", "area limit redirect=None decimal=2");
run("Set Scale...", "distance=0 known=0 pixel=1 unit=pixel global");
run("Colors...", "foreground=white background=black selection=magenta");
run("Smooth", "stack");
run("Split Channels");
selectImage(1);
rename("C1");
selectImage(2);
rename("C2");

// Generate a Channel 1 image with subtracted background
selectImage("C1");
setAutoThreshold("Triangle dark");
run("Create Selection");
run("Make Inverse");
        getStatistics(area, mean);
          C1bg=mean;
                    print("C1-background: ",C1bg);
```

```
        run("Select None");
        run("Subtract...", "value="+C1bg);
setAutoThreshold("Triangle dark");
run("Create Selection");
setBackgroundColor(0, 0, 0);
run("Clear Outside");
run("Enhance Contrast...", "saturated=0.1 equalize");


// Generate a Channel 2 image with subtracted background
selectImage("C2");
setAutoThreshold("Triangle dark");
run("Create Selection");
run("Make Inverse");
          getStatistics(area, mean);
            C2bg=mean;
                        print("C2-background: ",C2bg);
        run("Select None");
        run("Subtract...", "value="+C2bg);
setAutoThreshold("Triangle dark");
run("Create Selection");
setBackgroundColor(0, 0, 0);
run("Clear Outside");
run("Enhance Contrast...", "saturated=0.1 equalize");

run("Coloc 2", "channel_1=[C1] channel_2=[C2] roi_or_mask=<None>
threshold_regression=Bisection display_shuffled_images li_icq
spearman's_rank_correlation manders'_correlation kendall's_tau_rank_correlation
2d_instensity_histogram psf=3 costes_randomisations=10");

selectWindow("C1");
run("Select None");
run("Duplicate...", "title=thresholded1");
setAutoThreshold("MaxEntropy dark");
run("Duplicate...", "title=threshold1");
setAutoThreshold("MaxEntropy dark");
run("Convert to Mask");
run("Watershed");
run("Analyze Particles...", "size=25-Infinity circularity=0.00-1.00 show=Masks
summarize");
rename("Mask1");
selectWindow("threshold1");
close();

selectWindow("C2");
run("Select None");
run("Duplicate...", "title=thresholded2");
setAutoThreshold("MaxEntropy dark");
run("Duplicate...", "title=threshold2");
setAutoThreshold("MaxEntropy dark");
run("Convert to Mask");
```

```
run("Watershed");
run("Analyze Particles...", "size=25-Infinity circularity=0.00-1.00 show=Masks
summarize");
rename("Mask2");
selectWindow("threshold2");
close();

imageCalculator("Max create", "Mask1","Mask2");
rename("combination");
setAutoThreshold("MaxEntropy");
run("Analyze Particles...", "size=25-Infinity summarize add");

imageCalculator("AND create", "Mask1","Mask2");
rename("colocalization");
setAutoThreshold("MaxEntropy");
run("Analyze Particles...", "size=25-Infinity circularity=0.00-1.00 show=Nothing
summarize");
run("Create Selection");

selectImage("C1");
run("RGB Color");
selectImage("C2");
run("RGB Color");
run("Duplicate...", "title=merged");
setPasteMode("Blend");
selectImage("C1");
run("Copy");
selectImage("merged");
run("Paste");
run("Restore Selection");
run("Draw", "slice");
run("Colors...", "foreground=blue background=black selection=magenta");
run("ROI Manager...");
roiManager("Draw");
run("Colors...", "foreground=white background=black selection=magenta");
selectWindow("ROI Manager");
run("Close");

selectWindow("thresholded1");
close();
selectWindow("Mask1");
close();
selectWindow("thresholded2");
close();
selectWindow("Mask2");
close();
selectImage("combination");
close();
selectImage("colocalization");
close();
```

```
run("Images to Stack", "name=Stack title=[] use");
run("Make Montage...", "columns=3 rows=1 scale=1 border=3 font=12 label use");
```

Supplementary information 2: Fiji/ImageJ Macro with automated Default threshold

**Fiji – ImageJ Macro for objected corrected colocalization analyses**

**with automated Default threshold**

```
// The macro uses two channels (termed C1, C2) to determine colocalization
// From each channel a background is subtracted after thresholding
// with a triangel algorithm; outside the thresholded objects the image is cleared
// Then the channels are equalized to the intensity range so that the following thresholding
// finds equal conditions for both channels (in case of different intensities)
// A Default threshold is then used for object identification for each channel.
// Objects are binarized followed by a watershed segmentation
// overlapping objects are determined (colocalization)
// A combination of objects is generated (combination)
// area measurements of colocalization and combination serve to determine
// the fraction of colocalization

run("Set Measurements...", "area limit redirect=None decimal=2");
run("Set Scale...", "distance=0 known=0 pixel=1 unit=pixel global");
run("Colors...", "foreground=white background=black selection=magenta");
run("Smooth", "stack");
run("Split Channels");
selectImage(1);
rename("C1");
selectImage(2);
rename("C2");

// Generate a Channel 1 image with subtracted background
selectImage("C1");
```

```
setAutoThreshold("Triangle dark");
run("Create Selection");
run("Make Inverse");
        getStatistics(area, mean);
         C1bg=mean;
                 print("C1-background: ",C1bg);
     run("Select None");
     run("Subtract...", "value="+C1bg);
setAutoThreshold("Triangle dark");
run("Create Selection");
setBackgroundColor(0, 0, 0);
run("Clear Outside");
run("Enhance Contrast...", "saturated=0.1 equalize");


// Generate a Channel 2 image with subtracted background
selectImage("C2");
setAutoThreshold("Triangle dark");
run("Create Selection");
run("Make Inverse");
        getStatistics(area, mean);
         C2bg=mean;
                 print("C2-background: ",C2bg);
     run("Select None");
     run("Subtract...", "value="+C2bg);
setAutoThreshold("Triangle dark");
run("Create Selection");
setBackgroundColor(0, 0, 0);
run("Clear Outside");
run("Enhance Contrast...", "saturated=0.1 equalize");
```

```
run("Coloc 2", "channel_1=[C1] channel_2=[C2] roi_or_mask=<None>
threshold_regression=Bisection display_shuffled_images li_icq spearman's_rank_correlation
manders'_correlation kendall's_tau_rank_correlation 2d_instensity_histogram psf=3
costes_randomisations=10");

selectWindow("C1");
run("Select None");
run("Duplicate...", "title=thresholded1");
setAutoThreshold("Default dark");
run("Duplicate...", "title=threshold1");
setAutoThreshold("Default dark");
run("Convert to Mask");
run("Watershed");
run("Analyze Particles...", "size=25-Infinity circularity=0.00-1.00 show=Masks summarize");
rename("Mask1");
selectWindow("threshold1");
close();

selectWindow("C2");
run("Select None");
run("Duplicate...", "title=thresholded2");
setAutoThreshold("Default dark");
run("Duplicate...", "title=threshold2");
setAutoThreshold("Default dark");
run("Convert to Mask");
run("Watershed");
run("Analyze Particles...", "size=25-Infinity circularity=0.00-1.00 show=Masks summarize");
rename("Mask2");
selectWindow("threshold2");
close();
```

```
imageCalculator("Max create", "Mask1","Mask2");
rename("combination");
setAutoThreshold("Default");
run("Analyze Particles...", "size=25-Infinity summarize add");

imageCalculator("AND create", "Mask1","Mask2");
rename("colocalization");
setAutoThreshold("Default");
run("Analyze Particles...", "size=25-Infinity circularity=0.00-1.00 show=Nothing summarize");
run("Create Selection");

selectImage("C1");
run("RGB Color");
selectImage("C2");
run("RGB Color");
run("Duplicate...", "title=merged");
setPasteMode("Blend");
selectImage("C1");
run("Copy");
selectImage("merged");
run("Paste");
run("Restore Selection");
run("Draw", "slice");
run("Colors...", "foreground=blue background=black selection=magenta");
run("ROI Manager...");
roiManager("Draw");
run("Colors...", "foreground=white background=black selection=magenta");
selectWindow("ROI Manager");
run("Close");
```

```
selectWindow("thresholded1");
close();
selectWindow("Mask1");
close();
selectWindow("thresholded2");
close();
selectWindow("Mask2");
close();
selectImage("combination");
close();
selectImage("colocalization");
close();

run("Images to Stack", "name=Stack title=[] use");
run("Make Montage...", "columns=3 rows=1 scale=1 border=3 font=12 label use");
```

Supplementary information 3 *Fiji/ImageJ* Macro with manual user-defined threshold

**_Fiji – ImageJ_ Macro for objected corrected colocalization analyses with manual user-defined threshold**

```
// The macro uses two channels (termed C1, C2) to determine colocalization
// From each channel a background is subtracted after thresholding
// with a triangel algorithm; outside the thresholded objects the image is cleared
// Then the channels are equalized to the intensity range so that the following thresholding
// finds equal conditions for both channels (in case of different intensities)
// A manual threshold is then used for object identification for each channel.
// Objects are binarized followed by a watershed segmentation
// overlapping objects are determined (colocalization)
// A combination of objects is generated (combination)
// area measurements of colocalization and combination serve to determine
// the fraction of colocalization

run("Set Measurements...", "area limit redirect=None decimal=2");
run("Set Scale...", "distance=0 known=0 pixel=1 unit=pixel global");
run("Colors...", "foreground=white background=black selection=magenta");

selectImage(1);
rename("C1");
selectImage(2);
rename("C2");


// Generate a Channel 1 image with subtracted background
selectImage("C1");
setAutoThreshold("Triangle dark");
run("Create Selection");
run("Make Inverse");
        getStatistics(area, mean);
         C1bg=mean;
                print("C1-background: ",C1bg);
       run("Select None");
       run("Subtract...", "value="+C1bg);
setAutoThreshold("Triangle dark");
run("Create Selection");
setBackgroundColor(0, 0, 0);
run("Clear Outside");
run("Enhance Contrast...", "saturated=0.1 equalize");


// Generate a Channel 2 image with subtracted background
selectImage("C2");
setAutoThreshold("Triangle dark");
run("Create Selection");
```

```
run("Make Inverse");
          getStatistics(area, mean);
           C2bg=mean;
                      print("C2-background: ",C2bg);
        run("Select None");
        run("Subtract...", "value="+C2bg);
setAutoThreshold("Triangle dark");
run("Create Selection");
setBackgroundColor(0, 0, 0);
run("Clear Outside");
run("Enhance Contrast...", "saturated=0.1 equalize");


selectWindow("C1");
run("Select None");
run("Duplicate...", "title=thresholded1");
run("Duplicate...", "title=threshold1");
run("Threshold...");
waitForUser("Set threshold and click OK")
run("Convert to Mask");
run("Watershed");
run("Analyze Particles...", "size=5-Infinity circularity=0.00-1.00 show=Masks summarize");
rename("Mask1");
selectWindow("threshold1");
close();


selectWindow("C2");
run("Select None");
run("Duplicate...", "title=thresholded2");

run("Duplicate...", "title=threshold2");
run("Threshold...");
waitForUser("Set threshold and click OK")
run("Convert to Mask");
run("Watershed");
run("Analyze Particles...", "size=5-Infinity circularity=0.00-1.00 show=Masks summarize");
rename("Mask2");
selectWindow("threshold2");
close();

imageCalculator("Max create", "Mask1","Mask2");
rename("combination");
run("Watershed");
setAutoThreshold("Default");
run("Analyze Particles...", "size=5-Infinity circularity=0.00-1.00 show=Nothing summarize");

imageCalculator("AND create", "Mask1","Mask2");
```

```
rename("colocalization");
setAutoThreshold("Default");
run("Analyze Particles...", "size=5-Infinity circularity=0.00-1.00 show=Nothing summarize");
run("Create Selection");

selectImage("C1");
run("RGB Color");
selectImage("C2");
run("RGB Color");
run("Duplicate...", "title=merged");
setPasteMode("Blend");
selectImage("C1");
run("Copy");
selectImage("merged");
run("Paste");
run("Restore Selection");
run("Draw", "slice");


selectWindow("thresholded1");
close();
selectWindow("Mask1");
close();
selectWindow("thresholded2");
close();
selectWindow("Mask2");
close();
selectImage("combination");
close();
selectImage("colocalization");
close();

run("Images to Stack", "name=Stack title=[] use");
run("Make Montage...", "columns=3 rows=1 scale=0.5 font=12 label use");
```

Suppl. Figure 1: Test of the analysis procedure with an independent data set

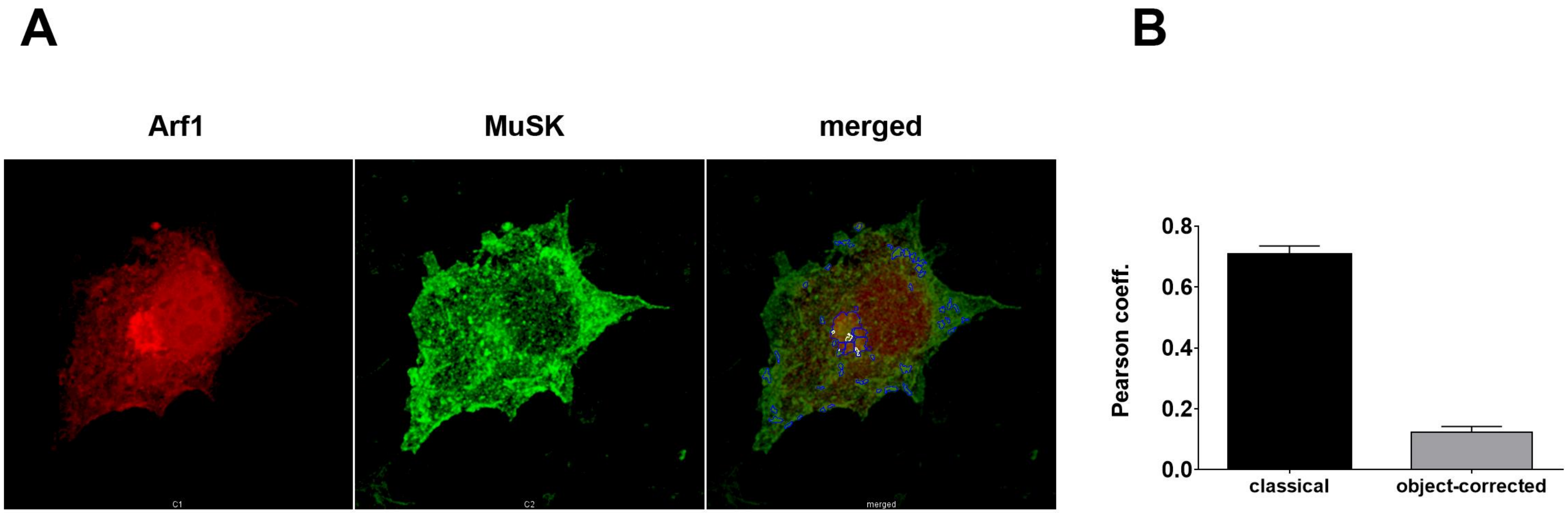


**Suppl. Figure 1: Test of the analysis procedure with an independent data set:** A) Fluorescence microscopy of Arf1 (ADP-Ribosyloation Factor 1) and MuSK (Muscle-specific kinase). which are known to localize to different compartments (Luiskandl S, Woller B, Schlauf M, Schmid JA, Herbst R: Endosomal trafficking of the receptor tyrosine kinase MuSK proceeds via clathrin-dependent pathways, Arf6 and actin. The FEBS journal 2013, 280(14):3281-3297). Colocalization analysis was done as described in the main text. The merge image contains a blended combination of both fluorescence channels. B) Quantification of fluorescence colocalization using a classical Pearson correlation coefficient or the object-corrected Pearson coefficient as proposed in this manuscript (mean +/- SEM, n=9).
